# Loss of Ethanolamine Utilization in Enterococcus faecalis Increases Gastrointestinal Tract Colonization

**DOI:** 10.1128/mBio.00790-18

**Published:** 2018-05-08

**Authors:** Karan Gautam Kaval, Kavindra V. Singh, Melissa R. Cruz, Sruti DebRoy, Wade C. Winkler, Barbara E. Murray, Danielle A. Garsin

**Affiliations:** a Department of Microbiology and Molecular Genetics, McGovern Medical School, University of Texas Health Science Center at Houston, Houston, Texas, USA; b Department of Internal Medicine, McGovern Medical School, University of Texas Health Science Center at Houston, Houston, Texas, USA; c Department of Cell Biology and Molecular Genetics, University of Maryland, College Park, Maryland, USA; d The UT Center for Antimicrobial Resistance and Microbial Genomics, McGovern Medical School, University of Texas Health Science Center at Houston, Houston, Texas, USA; UT Southwestern Medical Center Dallas

**Keywords:** *Enterococcus*, ethanolamine, intestinal colonization

## Abstract

Enterococcus faecalis is paradoxically a dangerous nosocomial pathogen and a normal constituent of the human gut microbiome, an environment rich in ethanolamine. E. faecalis carries the *eut* (ethanolamine utilization) genes, which enable the catabolism of ethanolamine (EA) as a valuable source of carbon and/or nitrogen. EA catabolism was previously shown to contribute to the colonization and growth of enteric pathogens, such as Salmonella enterica serovar Typhimurium and enterohemorrhagic Escherichia coli (EHEC), in the gut environment. We tested the ability of *eut* mutants of E. faecalis to colonize the gut using a murine model of gastrointestinal (GI) tract competition and report the surprising observation that these mutants outcompete the wild-type strain.

## IMPORTANCE

Some bacteria that are normal, harmless colonizers of the human body can cause disease in immunocompromised patients, particularly those that have been heavily treated with antibiotics. Therefore, it is important to understand the factors that promote or negate these organisms’ ability to colonize. Previously, ethanolamine, found in high concentrations in the GI tract, was shown to promote the colonization and growth of bacteria associated with food poisoning. Here, we report the surprising, opposite effect of ethanolamine utilization on the commensal colonizer E. faecalis, namely, that loss of this metabolic capacity made it a better colonizer.

## OBSERVATION

Ethanolamine (EA) is a compound found in the gastrointestinal (GI) tract at concentrations of 1 to 2 mM (1, 2). Interestingly, the genes that code for the catabolism of this compound, the *eut* (ethanolamine utilization) genes, are associated with gut pathogens, including species of *Escherichia, Salmonella*, *Clostridium*, and *Listeria* (3). In species such as Salmonella enterica serovar Typhimurium and enterohemorrhagic Escherichia coli (EHEC), mutants lacking the ability to sense and/or catabolize EA are outcompeted by wild-type strains in the gastrointestinal tract (1, 2, 4) or display neutral colonization efficacy in the case of Clostridium difficile (5).

Enterococcus faecalis also encodes the *eut* genes and is found in the GI tract, but unlike the above-mentioned examples, it is considered a commensal colonizer rather than a gut pathogen of healthy people. However, the presence of E. faecalis in the GI tract can serve as a source of nosocomial infection (6). Therefore, understanding the factors that promote the colonization and growth of E. faecalis in the gut is important for the development of strategies to mitigate these infections.

**FIG 1:**
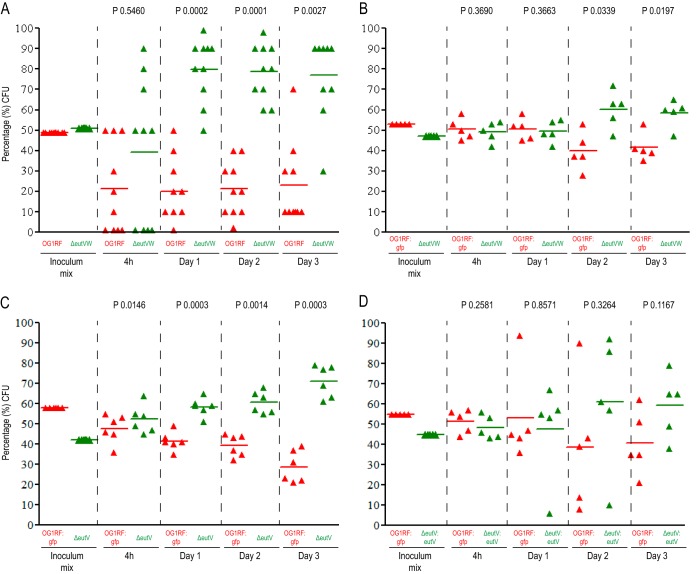
Competitive colonization of the gastrointestinal tract by the E. faecalis wild type and mutant strains following combined inoculation. Percentages of CFU of E. faecalis strains from the initial inoculum mix and from stool samples collected 4 h and 1, 2, and 3 days after mixed inoculation of E. faecalis OG1RF and the Δ*eutVW* mutant (AR2) (A), OG1RF::*gfp* and the Δ*eutVW* mutant (AR2) (B), OG1RF::*gfp* and the Δ*eutV* mutant (SD54) (C), and OG1RF::*gfp* and the Δ*eutV*::P_*eutS*_ *eutV* mutant (SD209) (D). In panel A, 10 CFU per mouse per time point were randomly picked and PCR screened to confirm the strains’ identities. For panels B, C, and D, >100 CFU were scored for GFP fluorescence per mouse per time point. An unpaired *t* test with Welch’s correction for the percentages of bacteria recovered from the stool samples versus the amounts in the initial inoculum mixture was used to calculate the *P* values.

To investigate the role of EA in GI tract colonization, we first tested OG1RF, a wild-type strain of E. faecalis commonly used in animal models (7), and an isogenic Δ*eutVW* mutant that lacks the two-component system that senses EA (8). EutW is a sensor histidine kinase that, upon binding EA, autophosphorylates and then phosphotransfers to EutV the cognate RNA binding response regulator that activates *eut* gene expression by an antitermination mechanism (8, 9). The Δ*eutVW* mutant does not express the *eut* genes and cannot utilize EA (8, 9). The strains were competed in a murine GI tract model in which the animals were pretreated with an antibiotic cocktail designed to reduce the endogenous flora and facilitate E. faecalis colonization (10). To measure levels of colonization, fecal pellets were collected at 1, 2, and 3 days postinoculation and plated for numbers of CFU on medium selective for enterococci. Fecal pellets were also collected at 4 h to check for a spike in CFU, indicating a failure to colonize. Ten colonies/mouse/time point were screened by PCR for the presence of the deletion that marked the Δ*eutVW* mutant strain. As shown in Fig. 1A, the Δ*eutVW* mutant significantly outcompeted the wild type at all colonization time points.

Because screening the colonies by PCR was labor-intensive, we decided to repeat the experiment using a previously generated marked strain of OG1RF that constitutively expresses *gfp* present as a chromosomal insertion (11). By exposing the plated colonies from the fecal pellets to a fluorescent stereoscope, the presence of green fluorescent protein (GFP) could easily be discerned, allowing hundreds of colonies from each mouse to be screened. To ensure that GFP expression did not deleteriously affect the fitness of our wild-type strain, it was competed against the parent strain lacking the marker; a significant difference was not observed (see Fig. S1 in the supplemental material). We repeated the competition experiment with the Δ*eutVW* mutant versus the wild type and again observed the mutant outcompeting the wild-type strain (Fig. 1B). Note that the inoculum of the Δ*eutVW* mutant was purposely kept lower than that of the wild type so as not to create a bias toward the mutant, since the first experiment indicated that it was more fit.

10.1128/mBio.00790-18.1 FIG S1  Colonization of the gastrointestinal tract by OG1RF versus OG1RF::*gfp* following mixed inoculation. Numbers of CFU (A) and calculated percentages of CFU (B) of E. faecalis strains from stool samples collected at 4 h and 1, 2, and 3 days after combined inoculation. More than 100 CFU were scored for GFP fluorescence per mouse per time point. An unpaired *t* test with Welch’s correction for the percentages of bacteria recovered from the stool samples versus the amounts in the initial inoculum mixture was used to calculate the *P* values. Download FIG S1, EPS file, 0.7 MB. Copyright © 2018 Kaval et al. 2018 Kaval et al. This content is distributed under the terms of the Creative Commons Attribution 4.0 International license.

Considering that EA utilization mutants in other studied bacterial species tended to be less fit in the GI tract, our results showing that an E. faecalis mutant was modestly more fit were surprising. To confirm the finding, we tested a different mutant containing an in-frame deletion of only *eutV*. Also examined was a complement of this strain, able to induce *eut* gene expression to levels just slightly lower than those induced by the wild type (11). As shown in Fig. 1C, the Δ*eutV* mutant outcompeted the wild type at all time points examined, in contrast to the complemented strain (Fig. 1D).

The *eut* genes in EHEC and *S*. Typhimuirum are regulated by a mechanism different from that found in E. faecalis. These bacteria encode an EA-sensing transcriptional activator called EutR (12). In addition to the *eut* genes, EutR was found to bind promoters and directly activate the expression of some virulence factors in these pathogens (13, 14). Because of this additional activity, loss of the regulator resulted in a more severe phenotype than loss of just ethanolamine catabolism, depending on the specifics of the host environment (4). We wondered whether the phenotype observed with the loss of EutV was related solely to its role in regulating genes related to EA catabolism or whether it, like EutR, perhaps had a broader role.

**FIG 2:**
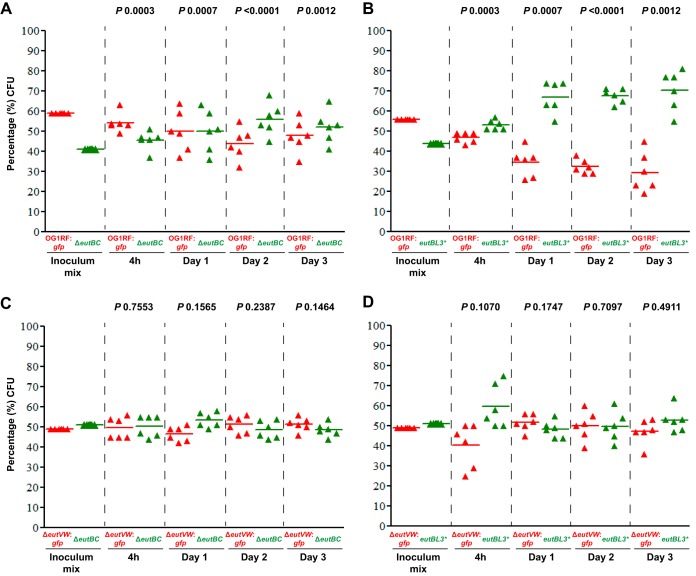
Murine gastrointestinal tract colonization by different E. faecalis strains following mixed inoculation. Percentages of CFU of E. faecalis strains from the initial inoculum mix and from stool samples collected 4 h and 1, 2, and 3 days after mixed inoculation of E. faecalis OG1RF::*gfp* (SD234) with the Δ*eutBC* strain (EFKK4) (A), OG1RF::*gfp* with the *eutB* mutant (eutBL3*) (EFKK12) (B), the Δ*eutVW*::*gfp* mutant (EFKK1) with the Δ*eutBC* mutant (C), and the Δ*eutVW*::*gfp* mutant with the *eutB* mutant (eutBL3*) (D). More than 100 CFU were scored for GFP fluorescence per mouse per time point. *P* values were calculated using an unpaired *t* test with Welch’s correction for the percentages of bacteria recovered from the fecal samples versus the amounts in the initial mixed inoculum.

To address this question, we created an in-frame deletion, Δ*eutBC*, to remove the genes encoding the two subunits of the ethanolamine ammonia lyase, which carries out the first reaction in the breakdown of EA. Many attempts to generate a complement of this mutant failed. Therefore, we created a different, independent mutant by generating a stop codon in *eutB*, called eutBL3*. Neither the in-frame deletion nor the stop mutant disrupted expression of the downstream *eut* structural genes, as bacterial microcompartment formation was still observed in both mutants (Fig. S2). Both mutants significantly outcompeted the wild type on days 2 and 3, with the stop codon mutant also displaying significant differences on day 1 (Fig. 2A and B). To test whether loss of EA sensing resulted in a greater fitness increase than loss of EA catabolism, we competed a Δ*eutVW* strain marked with GFP against the Δ*eutBC* strains (Fig. 2C and D). A significant difference was not observed for either strain pair at any time point. The data support the conclusion that EA catabolism alone contributes to the phenotype. These data fit former observations that the regulatory sequences recognized by E. faecalis EutV are found only in the *eut* transcripts (9).

10.1128/mBio.00790-18.2 FIG S2  Bacterial microcompartment formation in E. faecalis mutant strains. Transmission electron micrographs showing the formation of bacterial microcompartments (indicated by white arrows) in E. faecalis OG1RF (wild type), the Δ*eutBC* mutant (EFKK4), and the *eutB* mutant (eutBL3*) (EFKK12), when grown under inducing conditions. Download FIG S2, EPS file, 3.5 MB. Copyright © 2018 Kaval et al. 2018 Kaval et al. This content is distributed under the terms of the Creative Commons Attribution 4.0 International license.

10.1128/mBio.00790-18.3 TEXT S1  Supplemental materials and methods. Download TEXT S1, DOCX file, 0.03 MB. Copyright © 2018 Kaval et al. 2018 Kaval et al. This content is distributed under the terms of the Creative Commons Attribution 4.0 International license.

In conclusion, we present the surprising observation that EA catabolism in E. faecalis modestly reduces GI tract colonization efficiency, in contrast to that observed for three gut pathogens (1, 2, 4, 5). The difference might arise from lifestyle, as E. faecalis is a normal gut commensal in mammals; in Caenorhabditis elegans, which E. faecalis kills, a *eut* mutant was attenuated (15). A deeper understanding awaits further investigation.
