# Supplementary material for: Loss of Ethanolamine Utilization in Enterococcus faecalis Increases Gastrointestinal Tract Colonization
Source: mBio. 2018 May 8;9(3):e00790-18. doi: 10.1128/mBio.00790-18 (PMC5941071; doi:10.1128/mBio.00790-18)
Supplement: TEXT S1 [file mbo003183882s1.docx]

**MATERIALS AND METHODS**

**Bacterial strains and media**

All the bacterial strains utilized in this study are listed in Table S1. Growth media were procured from BD Bacto™ or BD Difco™, and reagents and chemicals from Thermo Fisher Scientific and Sigma, unless otherwise stated. *E. coli* strains were aerobically cultured in Luria Bertani (LB) broth with shaking on a Thermo Scientific culture rotator at a 45° angle in a 37°C incubator. 300 µg/ml of erythromycin and 100 µg/ml of spectinomycin (final concentration) were added when required. *E. faecalis* strains were also aerobically cultured in the same manner as described above in Brain Heart Infusion (BHI) broth unless otherwise mentioned. 50 µg/ml of erythromycin and 100 µg/ml rifampicin was used for selection pressure.

**Primers, DNA manipulations and sequencing**

All the primers utilized in this study are listed in Table 1. Primers were purchased from Sigma-Aldrich (St. Louis, MO) and Integrated DNA Technologies (Coralville, IA). All PCR reactions were performed using Phusion® High-Fidelity DNA polymerase (NEB, Ipswich, MA). Restriction enzymes and T4 DNA ligase for restriction-based cloning were procured from NEB and Promega (Madison, WI), respectively, and were used as per the manufacturer’s instructions. NEBuilder® HiFi DNA Assembly Master Mix (NEB) was used for all restriction-free assemblies. Purification of PCR products and extraction of plasmids was performed using kits from Qiagen (Hilden, Germany) as per the manufacturer’s guidelines. Genewiz (South Plainfield, NJ) performed all the sequencing of the constructs.

**Construction of plasmids and allelic exchange**

All plasmids used in this study have been listed in Table S1. For the construction of plasmid pKK1, primers KK5 and KK6 were used to amplify a ~800 bp fragment directly upstream of the *eutB* ORF from *E. faecalis* OG1RF genomic DNA. Similarly, primers KK7 and SD181 were employed to amplify a ~800 bp fragment directly downstream of the *eutC* ORF. Primers KK5 and KK181 were used to splice the upstream and downstream fragments together by overlap extension PCR, the product of which was inserted into pCR2.1 via TA cloning and transformed into chemically competent *E. coli* TOP10. This spliced fragment was then digested with the restriction enzymes, PstI and NotI, and subcloned into a similarly digested pCJK47 plasmid backbone and electroporated into electrocompetent *E. coli* EC1000 to give the plasmid, pKK3. Plasmid pKK18 was constructed, firstly, by amplifying two fragments from *E. faecalis* OG1RF genomic DNA using primers KK110 and K111, and KK112 and KK113, respectively. The pCJK47 plasmid backbone was PCR-linearized with primers KK108 and KK109. The two fragments and the linearized plasmid were then fused together via using the NEBuilder® HiFi DNA Assembly kit as per the manufacturer's instructions and transformed into EC1000 by electroporation. The plasmids as mentioned above were then transformed into the conjugative *E. faecalis* strain CK111 by electroporation. Conjugation of these strains namely, EFKK2 and EFKK11, with OG1RF, followed by allelic exchange as described in Kristich et al. (1), resulted in the respective *E. faecalis eutBC* mutant strains EFKK4 and EFKK12. The same allelic replacement approach was used construct strain EFKK1 using strains SD233 and AR2.

**Murine Gastrointestinal tract colonization model**

Competitive colonization experiments were performed following our previously published methods (2). Pre-approved protocol and guidelines laid out by the Animal Welfare Committee at the University of Texas Health Science Center at Houston were followed. Briefly, the experiments were performed using 6-week-old female ICR mice from Envigo. Mice were screened for presence of rifampicin resistant enterococci in fecal pellets, before and after decolonization, by plating the homogenized, serially diluted samples on Bile Esculin Agar (BEA) containing 100 µg/ml rifampicin (BEA-Rf). No Rf resistant colonies were observed in any of the mice. Immediately, mice were placed on decolonization antibiotic regimen for 4 days consisting of 1 mg/ml of gentamicin in the drinking water and subcutaneous administration of 2.4 mg/day/mouse of clindamycin. Bacterial gavages were performed 24 hours after the last antibiotic dose in order to allow the elimination of the antibiotics. The strains were administered in combination with a saline suspension containing approximately 10^9^-10^10^ CFUs. As the preliminary experiment (Figure 1A) indicated a competitive colonization advantage for the *E. faecalis eut* mutant strain, *eut* mutants in the subsequent experiments were administered at a slightly lower CFU count as compared to the reference strain in the combined inoculum. The fresh stool pellets (1 – 2 per mouse) were extracted by gentle abdominal massage and were collected directly into sterile preweighed Eppendorf tubes at 4h as well as days 1, 2 and three post-inoculation. The samples were then weighed, homogenized and serially diluted in 0.9% saline and plated on BEA-Rf plates. After incubation for 48 - 72 h at 37°C, 10 random colonies were picked PCR screened to confirm identity, as shown in the Figure 1A experiment, or, for all other experiments, the GFP-expressing *E. faecalis* strains were differentiated from the non-GFP-expressing strains under a Discovery V.8 fluorescent dissecting scope (Zeiss, Overkochen, Germany). >100 strain-specific CFUs/mouse/time point were screened in this manner. The CFUs/strain recovered at each time point were then expressed as a percentage of the total CFUs recovered at that time point.

**Transmission Electron Microscopy.**

*E. faecalis* strains OG1RF, EFKK4 and EFKK12 were grown in the presence of EA and AdoCbl to induce the expression of the *eut* genes, followed by staining and thin-sectioning for transmission electron microscopy as previously described in DebRoy et al. (3). The JEOL 1200 transmission electron microscopy system equipped with a Gatan 2k x 2k CCD camera was used for image acquisition.

**Statistical Analysis**

Statistical Analyses were done as described in Montealegre et al. (2) using GraphPad Prism, v7.0a (GraphPad Software, Inc., California). Briefly, a paired *t* test was used to analyze the data by comparing the percentage of each strain recovered at the different time points to the percentage in the inoculum.

**SUPPLEMENTARY REFERENCES**

1. Kristich CJ, Chandler JR, Dunny GM. 2007. Development of a host-genotype-independent counterselectable marker and a high-frequency conjugative delivery system and their use in genetic analysis of Enterococcus faecalis. Plasmid 57:131-44.

2. Montealegre MC, Singh KV, Murray BE. 2016. Gastrointestinal Tract Colonization Dynamics by Different Enterococcus faecium Clades. J Infect Dis 213:1914-22.

3. DebRoy S, Gebbie M, Ramesh A, Goodson JR, Cruz MR, van Hoof A, Winkler WC, Garsin DA. 2014. Riboswitches. A riboswitch-containing sRNA controls gene expression by sequestration of a response regulator. Science 345:937-40.
